# Nutritional Composition, Anti-Diabetic Properties and Identification of Active Compounds Using UHPLC-ESI-Orbitrap-MS/MS in *Mangifera odorata* L. Peel and Seed Kernel

**DOI:** 10.3390/molecules24020320

**Published:** 2019-01-16

**Authors:** Nur Fatimah Lasano, Azizah Haji Hamid, Roselina Karim, Mohd Sabri Pak Dek, Radhiah Shukri, Nurul Shazini Ramli

**Affiliations:** 1 Department of Food Science, Faculty of Food Science and Technology, Universiti Putra Malaysia, 43400 UPM Serdang, Selangor Darul Ehsan, Malaysia; nfatima9212@gmail.com (N.F.L.); azizahjan58@gmail.com (A.H.H.); mhdsabri@upm.edu.my (M.S.P.D.); 2 Department of Food Technology, Faculty of Food Science and Technology, Universiti Putra Malaysia, 43400 UPM Serdang, Selangor Darul Ehsan, Malaysia; rosaz@upm.edu.my (R.K.); radhiah@upm.edu.my (R.S.)

**Keywords:** *Kuini*, fruit, wastes, vitamins, minerals, phenolics, *α*-amylase, *α*-glucosidase

## Abstract

*Mangifera odorata* fruit, the hybrid forms between *M. indica* (mango) and *M. foetida* (*bacang*), has been shown to exhibit potential antioxidant activity, and the fruit waste could demonstrate functional and nutritional potential. In the present study, the nutritional composition (proximate, sugars, vitamins and minerals analyses), the anti-diabetic activities and phytochemical profile of *M. odorata* peel and seed kernel were investigated for the first time. The results indicated that seed kernel rich in fat, protein, carbohydrate, and ash while peel contained significantly greater amount of fiber, minerals, β-Carotene and ascorbic acid compared to seed kernel. The samples were then extracted using different solvents (acetone, ethanol, methanol at 60%, *v*/*v* and pure deionized water) and their anti-diabetic activities (*α*-amylase and *α*-glucosidase inhibition assay) were determined. Seed kernel had the lowest IC_50_ values for α-amylase and *α*-glucosidase inhibition assay in 60% ethanol and 60% acetone, respectively. Due to the toxic effect and high volatility of acetone, the ethanolic extracts of samples were further analyses for their phytochemical profile using high performance liquid chromatography-mass spectrometry (LC-MS) and ultra-high-performance liquid chromatography electrospray ionization orbitrap tandem mass spectrometry (UHPLC-ESI-Orbitrap-MS/MS). The most abundant compounds identified were phenolic acid, ellagic acid, and flavonoid. These findings suggest that *M. odorata* fruit wastes, especially the seed kernel possesses promising ability to be used as functional ingredient in the food industry.

## 1. Introduction

Diabetes mellitus is a leading cause of mortality and morbidity. Recent data showed that approximately 150 million people have diabetes worldwide, and this number may double by the year 2025 [1]. In Malaysia, the National Health and Morbidity Survey (NHMS) reported that the prevalence of diabetes has been increasing from 11.6% in 2006 to 17.5% in 2015 [2,3,4]. Moreover, Malaysia has been classified as the country with the highest prevalence of type 2 diabetes in Southeast Asia [5]. Complications associated with long duration of diabetes mellitus have been assumed to be related to chronically elevated glucose levels and subsequent oxidative stress [6]. The impact of diabetes on society is substantial. Like other chronic diseases, diabetes is an expensive disease both to an individual in terms of personal income and productivity from sickness and absenteeism, and to the community from the increasing and excessive burden on health care and rehabilitative facilities [7]. Hence, the direct and indirect economic cost of diabetes will continue to burden the country’s health care expenditure especially in low and middle-income countries. 

The mainstay of treatment for diabetes is oral hypoglycaemic agents [8]. Though modern pharmacotherapeutics like sulfonylureas and thiazolidines are effective in controlling hyperglycemia [9], it has prominent side effects such as hypoglycemia and atherogenesis [10]. Therefore, herbal formulations from natural sources are preferred to reduce the ill-effects of diabetes and its secondary complications not only due to the lesser side effects but also low cost which made them affordable by the low-income and those living in rural areas [11]. Realizing this, it is of great interest to conduct a study to identify plant-based herbal extracts as the primary components to diabetic management. Plants typically contain a mixture of compounds that may act individually, additively or in synergy to improve health. The hypoglycemic effects of some herbal extracts have been confirmed in human and animal models [11,12].

*Mangifera* species is a tropical plant that is proven to have pharmacological properties such as anti-diabetic [13], anti-cancer [14], anti-inflammatory [15], and the ability to attenuate kidney damage [16]. The mango fruit (*Mangifera indica* L.) and its wastes, is one of the extensively studied *Mangifera* species, owing to abundant nutrients and phytochemicals that benefits human health [17]. *M. odorata* is a hybrid between *M. indica* and *M. foetida* (*bacang*) [18]. It is one of the eight underutilized *Mangifera* species discovered in Malaysia [19]. The fruit flesh is orange-yellow in color, firm, fibrous, sour-sweet, and juicy with a pungent smell and taste of turpentine [20]. The fruit is highly nutritious where it contains higher protein and calcium compared to other *Mangifera* species, and had acceptable amounts of carotenoids [21,22].

Numerous studies reported the potent antioxidant activities of *M. odorata* fruit [23,24]; however, little information is available regarding the nutritional and functional properties of the fruit wastes (peel and seed kernel). Moreover, to the best of authors knowledge, studies on the effect of extraction solvents on the anti-diabetic activities and the recovery of functional components from different parts of *M. odorata* fruit were scarce. The fruit wastes could be excellent candidate for functional ingredients in food industry due to accumulation of valuable bioactive compounds as previously shown by mango seed and peel [25], which is believed to possess protective effects against oxidative stress-related diseases. Therefore, this study aimed at investigating the proximate composition, sugars, vitamins and minerals content of peel and seed kernel from *M. odorata* fruit. The samples were extracted using pure deionized water, 60% methanol, 60% ethanol, and 60% acetone, and their *α*-amylase and *α*-glucosidase inhibitory activities were evaluated. Furthermore, the major polyphenols in the peel and seed kernel were identified based on the optimized extracts using high performance liquid chromatography-mass spectrometry (LC-MS) and ultra-high-performance liquid chromatography electrospray ionization orbitrap tandem mass spectrometry (UHPLC-ESI-Ortbitrap-MS/MS). The results from this study could provide scientific evidences of health-promoting properties of *M. odorata* peel and seed kernel, and give insights to researchers to further explore the potential applications of fruits waste in food industry to eventually benefit the farmer, consumer and food scientist. 

## 2. Results and Discussion

### 2.1. Proximate Composition and Sugars Content in M. odorata Peel and Seed Kernel 

The proximate analysis of food includes moisture, crude protein, crude fat, carbohydrate, total dietary fiber and ash contents [26]. Data from the proximate analysis are important for the development of nutrition labeling and determination of quality and microbial safety of food [27,28]. Table 1 depicts the proximate composition of peel and seed kernel from *M. odorata* fruit. Results showed that peel contained greater moisture content (75.89 ± 0.70%) compared to seed kernel (50.03 ± 0.98%) suggested that peel may require drying process for better conservation of the product. However, the value was slightly lower in comparison with *M. odorata* pulp (79.5%) [29]. In addition, the moisture content of seed kernel was slightly higher than mango seed kernel (44.4%) [30]. Moreover, the results demonstrated that seed kernel contain significantly higher amount of crude protein, crude fat, carbohydrate and ash (*p* < 0.05) compared to peel and the fruit pulp [29]. On the other hand, fruit peel contains 50.94% total dietary fiber (34.78% soluble fibers and 16.16% insoluble fibers) compared to 24.75% total dietary fiber in seed kernel (*p* < 0.05). Interestingly, soluble fibers were not detected in the seed kernel. These results seem to be consistent with other research which found high amount of total dietary fibers in mango peel (51.2%) [31], and very low amount of crude fiber was found in mango kernel powder (9.33%) [32]. Previous studies reported that regular consumption of fibers, particularly soluble fibers, improved metabolic profiles and insulin in type 2 diabetes patients [33]. In their natural state, fruits and vegetables contain high amount of water and fiber, and very low level of fats (<0.5%), hence had low calories and energy density [34]. Accordingly, the present work showed seed kernel (208.50 kcal)/100g had more calorie than peel attributed to low level of moisture content and high amount of energy yielding nutrients, namely carbohydrate, fat and protein. It can thus be suggested that that the seed kernel of *M. odorata* fruit could be an important source of energy and essential nutrients to supplement human diet while peel provides significant sources of dietary fiber that may benefit diabetes patient.

Sugars content in the peel and seed kernel of *M. odorata* was quantified using HPLC and the results are shown in Table 1. It can be seen that there was no significant difference in sucrose content in both *M. odorata* peel and seed kernel (2.66 ± 0.36% and 2.66 ± 0.06%, respectively) (*p* > 0.05). Fructose content (2.25 ± 0.38%) was significantly higher in *M. odorata* peel while glucose content was significantly higher in the seed kernel (1.08 ± 0.27%) (*p* < 0.05). Overall, we observed that the predominant sugars in peel are sucrose and fructose while glucose only present in small amount. In contrary, sucrose is the main sugar found in the seed kernel, followed by glucose and fructose. This finding was consistent with other research which found that sucrose was the major sugar in the pineapple wastes [35]. Sucrose is a water-soluble disaccharide that is freely transported in different plant parts and efficiently stored energy compared to the simple monosaccharides, fructose and glucose. Therefore, these results are likely to be related to the role of sucrose to reserve energy for the seed development within the fruit [36]. In addition, a recent cross-sectional study involving diabetic patients found no convincing evidence between intake of fructose, glucose, and sucrose and β-cell function [37] suggesting that the consumption of these sugars may have little or no detrimental effect on diabetes. 

### 2.2. Minerals and Antioxidant Vitamins Content in M. odorata Peel and Seed Kernel

In addition to macronutrients, the minerals and vitamins content are also important for reliable nutritional information and necessary to evaluate diets for nutritional adequacy [38]. These essential nutrients are vital to maintain human health [39]. Mineral can be classified into major and trace elements depending on their concentration present and amount needed by human body. In the present study, six major minerals and six trace minerals were measured in *M. odorata* peel and seed kernel. The results are presented in Table 2. Minerals like potassium, calcium, sulphur, aluminum, manganese, iron, and boron were significantly higher in peel compared to seed kernel (*p* < 0.05). On the other hand, seed kernel was significantly high in magnesium, phosphorus, and zinc. The major mineral concentrations in different fruit parts ranged from 875.69 (seed kernel) to 1203.01 mg/100 g (peel) for potassium, 75.83 (peel) to 165.50 mg/100 g (seed kernel) for phosphorus, 141.48 (peel) to 391.83 mg/100 g (peel) for calcium, 147.75 (peel) to 166.88 mg/100 g (seed kernel) for magnesium, 43.03 (seed kernel) to 69.29 mg/100 g (peel) for sulphur, while sodium content ranged from 10.56 (seed kernel) and 10.77 mg/100 g (seed kernel). On the other hand, the trace minerals contents were found between 0.27 (seed kernel) to 0.76 mg/100 g (peel) for aluminum, 1.09 (seed kernel) to 2.25 mg/100 g (peel) for manganese, 1.08 (seed kernel) to 1.36 mg/100 g (peel) for iron, 0.65 (seed kernel) 0.78 mg/100 g (seed kernel) for copper, 1.12 (peel) to 1.55 mg/100 g (seed kernel) for zinc, and 0.87 (seed kernel) to 1.69 mg/100 g (peel) for boron. Irrespective of the fruit part, potassium was relatively the most abundant mineral element present in *M. odorata* fruit. 

To the best of the authors’ knowledge, this is the first report to demonstrate high minerals content in *M. odorata* wastes. These results could be related to the distribution of vascular tissue and sink characteristics of the plants [40,41,42]. Besides, the metabolic rate could also be a contributing factor. Tissues with higher metabolic rates (cells under the skin and in the core) may have higher requirements for nitrogen and phosphorus [43]. Moreover, rapidly expanding fruit parts, such as the cortical flesh of apples and melons, are unlikely to have high calcium concentrations. This is because their cell walls are elastic and less rigid than in non-expanding tissues. Tee et al. [29] studied several minerals and vitamin content in *M. odorata* pulp and found 9 mg/100 g and 13 mg /100 g of calcium and phosphorus, respectively. The values were much lower than the values reported in the present study. In brief, it was found that peel and seed kernel of *M. odorata* fruit could be a good source of minerals mainly potassium, calcium, and magnesium.

A vitamin is an organic molecule that cannot be produced by human body and must be supplied from the diet. Vitamins mostly present in fruits and vegetables and have important specific functions in normal body metabolism. It is usually grouped into fat and water-soluble molecules [39]. The present study focused on antioxidant vitamins which include vitamin A, C, and E. Besides, total carotenoid content (TCC) was also determined as it is the major pigment responsible for the fruit color. Table 2 shows the total carotenoid content (TCC) and antioxidant vitamins (Vitamin A, C, and E) of peel and seed kernel from *M. odorata* fruit. The data showed that peel had the highest TCC, β-Carotene, ascorbic acid and α-Tocopherol compared to seed kernel (*p* < 0.05). As expected from the color appearance, seed kernel had almost 10 times lower TCC than that of peel. Analysis of β-Carotene using HPLC showed similar trend whereby peel contained higher amount of β-Carotene compared to seed kernel (*p* < 0.05). It is interesting to highlight that β-Carotene content in *M. odorata* peel was higher than *M. indica* (35.67 μg /100g) and papaya (32.17 μg /100 g) [29]. When compared to whole *M. odorata* fruit, peel had the highest amount of TCC [21,22] while pulp had the highest amount of ascorbic acid [29]. However, finding on α-Tocopherol content from current study do not support the previous research whereby the seed of pumpkins showed the highest content of α-Tocopherol, followed by peel and pulp [44]. Data from the current work suggested that the peel of *M. odorata* fruit showed potential to be developed as functional food ingredient to supplement human diet since it is rich in carotenoid, ascorbic acid, and α-Tocopherol.

### 2.3. Effect of Different Solvent Extraction on Anti-Diabetic Activity of Peel and Seed Kernel from M. odorata Fruit

Post-prandial hyperglycemia commonly occurs in diabetic patient, and can be controlled by inhibition of the carbohydrate hydrolyzing enzymes (*α*-amylase and *α*-glucosidase) [45]. This can delay the absorption of glucose from the small intestine and eventually reduce blood glucose level [46]. In our work, the inhibitory activity of different parts of *M. odorata* extracted using different solvents against both enzymes were carried out. The lower IC_50_ value indicates greater inhibitory activity of the plant extract toward the enzymes. Acarbose was used as a positive control for *α*-amylase, while acarbose and quercetin were used as positive controls for *α*-glucosidase. The *α*-amylase inhibitory activity of different extracts of peel and seed kernel from *M. odorata* fruit were expressed as IC_50_ (mg/mL) values and are presented in Table 3. The seed kernel (2.67 to 9.83 mg/mL) exhibited the highest *α*-amylase inhibitory activity compared to the peel. The inhibition pattern for different solvent extracts of different fruit parts are significantly varied. For peel, ethanol extract showed the highest *α*-amylase inhibitory activity compared to other extracts. However, the seed kernel in water extract showed the most potent *α*-amylase inhibitory activity followed by ethanol, acetone, and methanol. The *α*-glucosidase inhibition assay also showed similar trend whereby the seed kernel (0.29 to 2.10 mg/mL) had the lowest IC_50_ value indicating highest inhibitory activity compared to peel (10.52 to 59.45 mg/mL). In terms of the solvent extraction, acetone extract showed lower IC_50_ value compared to other solvents for both fruit parts. In addition, the inhibitory activity against *α*-amylase and *α*-glucosidase of *M. odorata* seed kernel was more potent than *M. indica* seed extract [47]. As previously mentioned, all seed kernel extracts displayed potent inhibitory activity, better than acarbose and quercetin. Therefore, it could be suggested that the seed kernel from *M. odorata* can be an excellent candidate to use as natural hypoglycemic agent for the management of hyperglycemia in diabetes mellitus patient with lesser side effects than acarbose or other synthetic drugs. Taking together, these data suggested that 60% ethanol extracts and 60% acetone extracts displayed better anti-diabetic activity, but due to toxic effect and volatility of acetone, ethanol extract may have more potential for further investigation for ethanol is safe. Although water extract is the most economical solvent and environmental-friendly for large scale production, unfavorable results was obtained for *α*-glucosidase inhibition activity. Therefore, the identification of bioactive compounds was conducted using ethanol extracts of *M. odorata* peel and seed kernel. 

### 2.4. Screening of Untargeted Polyphenols in M. odorata Peel and Seed Kernel by Using LC-MS

**Figure 1:**
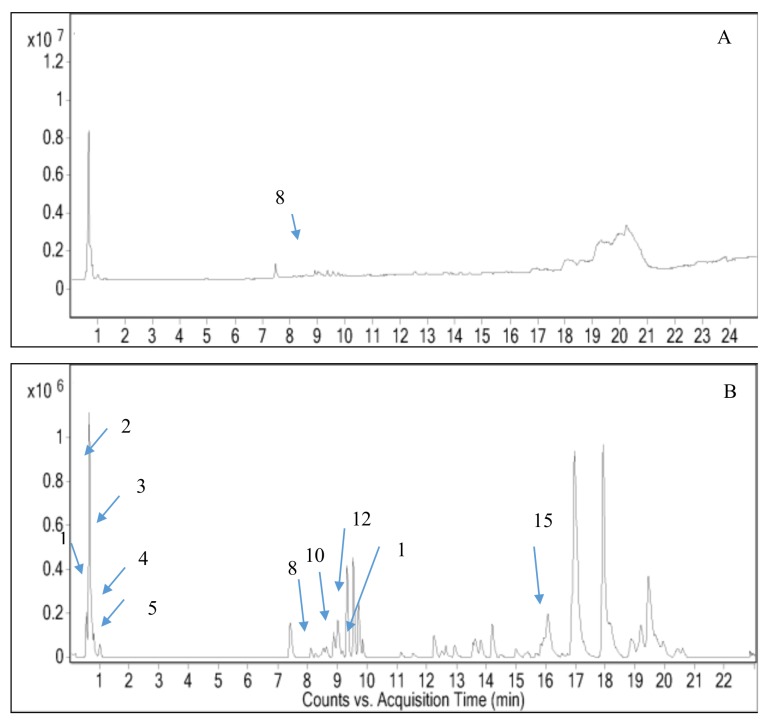
Total ion chromatogram from ethanol extract of *M. odorata* peel (**A**) negative mode and (**B**) positive mode.

**Figure 2:**
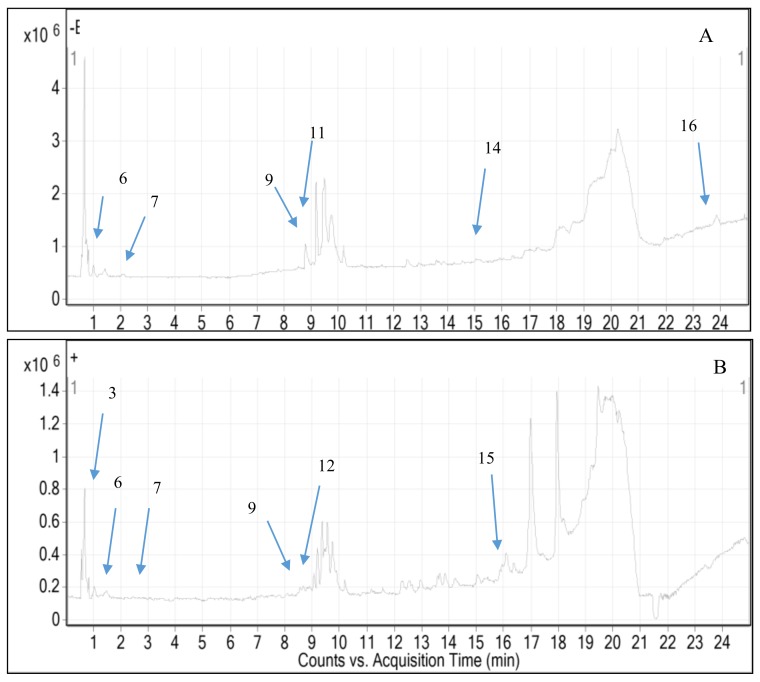
Total ion chromatogram from ethanol extract of *M. odorata* seed kernel (**A**) negative mode and (**B**) positive mode.

**Figure 3:**
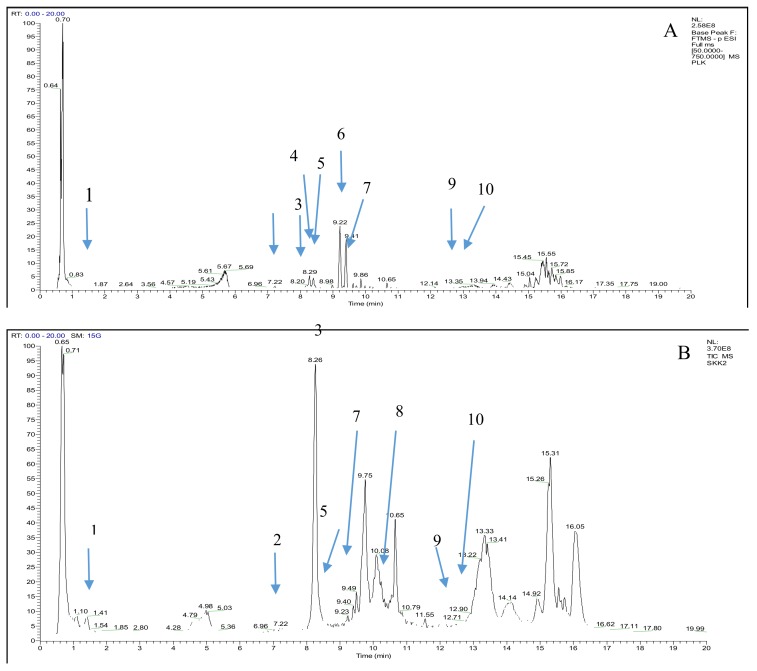
Total ion chromatogram from ethanol extracts of *M. odorata* (**A**) peel (**B**) seed kernel extracts.

Peel and seed kernel from mango are known to have high content of polyphenols that composed of phenolic acid, flavonoids, xanthones, tannins and others [48,49,50,51]. These compounds showed hypoglycemic effect through the inhibition of *α*-amylase and *α*-glucosidase activities [52]. Hence, we hypothesized that similar bioactive compounds might be presence in *M. odorata* wastes as they belong to the same species. The characterization of these compounds is an essential step for the utilization of different parts of *M. odorata* as food ingredients. In this regard, the use of chromatographic techniques able to characterize the bioactive compounds in the ethanolic extract of peel and seed kernel from *M. odorata*. A LC system coupled to quadrupole time-of-flight mass spectrometry (Q-TOF-MS) with dual ESI source of ethanol extracts of peel and seed kernel from *M. odorata* fruit was performed in both positive and negative ionization modes (Figure 1 and Figure 2). The data was processed using Molecular Feature Extraction (MFE) algorithm of the Agilent MassHunter Qualitative Analysis B.05.00 Workstation software to find likely compounds. The databases proposed the presence of 16 compounds, comprising phenolic glycosides, flavone, flavanone, and several phytochemicals that had mass match (5 ppm tolerance) in the database. Table 4 shows the selected compound identities and differentially compounds identified in the peel and seed kernel extracts from *M. odorata.* LC-MS screening data showed that the selected compounds in peel extract was more favorable in the positive ionization mode compared to seed kernel extract (Figure 3). This might be due to the presence of glycosides that are more sensitive in positive mode. On the other hand, beta-glucogallin, theogallin, 2-hydroxy-3,4-dimethoxybenzoic acid and mangiferin were the compounds that can be detected using both ionization modes. As shown in Table 4, peak 15 was identified as curcumenol. Curcumenol is the sesquiterpene that is known for its anti-inflammatory [53]. Moreover, quinic acid and 7,8,4′-trihydroxyflavanone were found in seed kernel and peel extracts, while dihydrocaffeic acid-3-*O*-glucuronide, *p*-salicyclic acid and dehydroascorbic acid were only present in peel extract. This finding is consistent with López-Cobo et al. [54] who reported quinic acid was major compound in *M. indica* seed husk, while *p*-salicyclic acid was detected in *M. indica* peel and seed extracts. It is interesting to highlight that mangiferin, apigenin 7-(2′′-*E*-*p*-coumaroylglucoside), (±)-naringenin and isovitexin were only found in the peel extract. Mangiferin is one of xanthone group that able to act as potent antioxidant and the activity is stronger than vitamin C and E [55]. There are many studies that successfully characterized mangiferin compound in different parts of mango [54,56,57]. In summary, the screening of untargeted compounds indicated that peel and seed kernel extract are rich sources of polyphenols which might contribute to anti-diabetic activity. Compounds such as mangiferin, naringenin, and isovitexin were only found in peel, while beta-glucogaliin, theogallin, 2-hydroxy-3,4-dimethoxybenzoic acid were only found in seed kernel. Further confirmation of the data is needed since different compounds may share similar molecular weight.

### 2.5. Identification and Confirmation of Targeted Polyphenols in the Peel and Seed Kernel of M. odorata Fruit by Using UHPLC-ESI-Ortbitrap-MS/MS

UHP-LC system coupled to Orbitrap-MS/MS with heated electrospray ionization (HESI) source of ethanol extracts of peel and seed kernel from *M. odorata* fruit led to separation and identification of the targeted polyphenols. These targeted compounds were the predominant polyphenols in mango fruit based on the literatures [58,59,60]. Therefore, 16 compounds that are most likely to contain in *M. odorata* wastes were selected. 16 authentic standards were used to identify and confirm the compound in the peel and seed kernel extract from *M. odorata* fruit (Figure S1). After comparing their retention times, [M − H]^−^ ion and MS/MS (MS^2^) spectra with the authentic standard, only 10 compounds were unambiguously detected in the sample extracts (Figure S2). The tentative assignment of these compounds is summarized along with the RT, observed *m*/*z*, MS and MS^2^ fragments (Table 5). The 10 compounds belong to six major group of polyphenols including phenolic acid, ellagic acid and flavonoids that is composed of flavanol, xanthone, flavonol and flavones. Table 6 shows relative abundance of peel and seed kernel extract from *M. odorata*. Peak 1 was identified as gallic acid [57,61,62]. Gallic acid has been previously described in several studies as the main polyphenol presence in mango peel and seeds [54,57,58,63,64]. Peak 3 was known as ethyl gallate [57,61,65]. Dorta et al. [57] revealed that ethyl gallate was the main compound in peel and seed of three mango varieties. Based on the MS and MS/MS data obtained from the authentic standard, peak 6 and 7 were identified as *p*-coumaric acid (*m*/*z* 162.03) and ellagic acid (*m*/*z* 300.99), respectively [66]. *p*-coumaric acid was found only in peel extract (Table 6). In contrast, similar compound has been detected in the peel of *M. pajang* [59] and kernel of *M. indica* [67,68]. Additionally, a low abundance of ellagic acid was found in both peel and seed kernel extracts (Table 6). This finding is consistent with that reported by Barreto et al. [58] and Dorta et al. [57] who revealed low amount of ellagic acid in peel and seed of mango. On the contrary, López-Cobo et al. [54] showed that ellagic acid was the predominant compound in seed of three cultivars of mango. The presence of flavanol was confirmed in the peel and seed kernel extracts from *M. odorata* fruit. Peaks 2 and 4 were determined as a catechin and epi-catechin based on the *m*/*z* value [61,69]. Both compounds can be found in peel extract while catechin showed the highest abundance in the seed kernel extract (Table 6). Similarly, the compounds have been successfully detected in different parts of *M. indica* and *M. pajang* [54,59,60,70]. Mangiferin was the only compound from xanthone group ([M − H]^−^ at *m*/*z* 421.07) (peak 5) [58,71]. This compound showed high abundance in the peel compared to seed kernel. Previous studies reported that mangiferin is the main polyphenols in mango bark, leaves, peel, and kernel [58]. The compound from peak 8 and 10 were identified as flavonol. Peak 10 is a myricetin and has [M − H]^−^ at *m*/*z* 317.03. It was detected only in the seed kernel extract by MS in small abundance. Myricetin was also detected in seed kernel of *M. indica* Waterlily [68]. The negative ion fragment of the peak 9 was identified as apigenin from flavone group ([M − H]^−^ at *m*/*z* 269.54) [72,73]. This compound was found in both peel and seed kernel extracts (Table 6). Apigenin was also detected in seed kernel of *M. indica* Waterlily as the main compounds after epigallocatechin and chlorogenic acid [68]. Peak 10 was identified as kaempferol which has [M − H]^−^ at *m*/*z* 285.04 and the fragment ions of peak 10 was in line with March et al. [74]. Table 6 shows low abundance of kaempferol in peel and seed kernel extract. Likewise, Ribeiro and Schieber [67] identified kaempferol in the kernel of *M. indica.* In conclusions, from the 16 targeted compounds in ethanolic extract of *M. odorata*, 10 compounds were identified by using UHPLC-MS/MS. The main phenolic group in the peel extract was composed of flavanol (catechin and epicatechin) and xanthone. Moreover, gallic acid, catechin and ethyl gallate were the predominant compounds in the seed kernel extract (Table 6). The detected compounds (phenolic acid, ellagic acid and flavonoids) have been shown to possess anti-diabetic properties due to the structural configuration of double bonds conjugated with 4-oxo function and hydroxyl groups [75]. Gallic acid was reported to exhibit *α*-glucosidase and *α*-amylase inhibitory activity (1.22 ug/mL and 1.09 ug/mL, respectively) [76] while apigenin showed inhibitory activity against *α*-amylase (8.1 mg/mL) [77]. Besides, myricetin, kaempferol, and ellagic acid also exhibited potent anti-diabetic activity through the inhibition of *α*-glucosidase and *α*-amylase activities [77,78]. The results of the present work suggested that the detected compounds may responsible for the potential anti-diabetic activity of *M. odorata* peel and seed kernel (Figure S3). It is important to highlight that the anti-diabetic activity of plant extracts is depending on the type of compounds, but not the amount presence [79]. Furthermore, it is important to consider the influence of geographical regions since it strongly affects the chemical composition of plant [80].

In brief, the present study revealed that *M. odorata* wastes can be good sources of bioactive compounds and can be potentially used as raw materials in various food applications. The utilization of fruit-by-products can contribute to zero waste and prevent environmental problems that will cause serious economic loss if not managed properly. In addition, the nutritional and functional values of *M. odorata* wastes provide evidences for the farmer to increase the cultivation and production of *M. odorata* fruit, and eventually contribute to economic activities and market growth from the valuable biowaste products. Similar approach has been successfully used in the production of balanced animal feed from mango seed powder (16, 24). The sustainability of the final products has yet to be explored. 

## 3. Materials and Methods 

### 3.1. Plants Material and Sample Preparation

*M. odorata* fruit was purchased from fresh market Serdang, Selangor. Voucher specimens of authentic *M. odorata* was acquired from Institute of Bioscience (Voucher No. SK 3179/17, Seri Kembangan, Malaysia). Cleaning of the fresh fruits were carried out before being processed. The peel and seed kernel of the fruit were separated followed by drying using freeze dry. Fine powder was obtained through grounding by using electronic grinder at room temperature (24 °C) and stored in airtight opaque at −20 °C until further analysis. Quartering technique was performed before extraction for sampling purposes.

### 3.2. Chemical and Reagents

Concentrated sulphuric acid, mix catalyst (96% sodium sulphate anhydrous, 3.5% cuprum sulphate sulphate, 0.5% selenium dioxide, sodium hydroxide (NaOH), sulphuric acid (H_2_SO_4_), boric acid, petroleum ether, *n*-hexane, iodine reagent, starch, potassium acetate, and potassium iodide were purchased from Merck (Kenilworth, New Jersey, USA). While, ethanol, methanol, acetone, acetic acid (C_2_H_4_O_2_), hydrochloric acid, 1 M phosphate buffer solution, alpha amylase from porcine pancreas, alpha glucosidase enzyme from Saccharomyces cerevisiae, PNPG (4-Nitrophenyl-α-d-glucopranoside), and acarbose were purchased and from Sigma (St. Louis, Missouri, USA). All the authentic standard (HPLC grade) from Sigma. The standards include ellagic acid, kaempferol, chlorogenic acid, apigenin, myricetin, *p*-coumaric acid, proto-catechuic acid, mangiferin, ethyl gallate, vanillic acid, *trans*-cinnamic acid, epicatechin, catechin, quercetin hydrate, gallic acid, quercetin, ascorbic acid, and catechin. 

### 3.3. Nutrition Composition

#### 3.3.1. Proximate Analysis

The moisture content was done according to procedure by Malaysian Standard (MS1191:1991) UDC 642.2:641.13 [81]. While, the ash value of the sample was measured by using standard AOAC (32.1.05) methods [26]. Tecator Manual [26] and Soxtec Manual [82] were used for the determination of crude protein and fat content, respectively. The carbohydrate content was calculated by subtracting the sum of total percent values of moisture, ash, protein, and fat from 100. The energy content of the sample was quantified using the factor of 4, 4, 9, and 2 of protein, carbohydrate, fat and dietary fiber, respectively [83]. Each of the nutrient contents was expressed as g per 100 g of fresh weight.

#### 3.3.2. Quantification of Simple Sugars by High Performance Liquid Chromatography

High Performance Liquid Chromatography (HPLC) on a Waters 600 HPLC instrument (Waters Co. Ltd., Milford, MA, USA) equipped with PhenoSpehere 5µm NH2 80A LC Column 250 × 4.6 mm (Merck, Kenilworth, New Jersey, USA) and Refractive index (Waters 410 Differential Refractometer) with WATERS 600 Controllers was used to determine the sugar content. Different concentrations of standard (fructose, glucose and sucrose) were prepared in the range of 0.25% to 2.50% (*w*/*v*). The sample undergone refluxing process and the solvent was removed using rotary evaporator. The extract was volume up to 25 mL before being used for analysis. Before injected to the system, all the samples (dried sample) and standards were filtered by using 25 mm 0.45 µm (N6) syringe filter. About 20 µL of standards and samples were injected into the system. HPLC analysis was performed in isocratic elution for 20 min in mobile phase of mixture acetonitrile: deionized water (80:20, *v*/*v*) with flow rate of 1.0 mL/min.

#### 3.3.3. Dietary Fibre

AOAC enzymatic-gravimetric official method (985.29) [26] were used to determine the total, insoluble and soluble dietary fibers (DF). The method used heat stable alpha-amylase, amyloglucosidase and protease treatment. Total DF was determined from the sum of insoluble DF and soluble DF.

#### 3.3.4. Minerals Content

The nitric-perchloric digestion was used to prepare the sample [84]. The atomic absorption spectrophotometer (AAS) flame (SpectrAA 110, Varian, Melbourne, Australia) was used to determine the major and trace element of the sample [85]. For each element, specific conditions of wavelength, slit, and mixing of the gases were set for the calibration of AAS. Calibration curves for each element were plotted using standard mineral diluted with deionized water. The results were expressed in mg per 100 g of sample on a dry weight basis (mg per 100 g DW). 

### 3.4. Antioxidant Vitamins

#### 3.4.1. Determination of Total Carotenoid Content

The determination of total carotenoid content was carried out based on the method by Khoo et al. [21]. Mixtures of freeze-dried sample (0.5 g) and 15 mL of hexane was vortexes and left for few minutes before being centrifuged at 3000 rpm for a one min. The clear hexane extract was collected and residue re-extracted until become colorless. Then, the hexane extract was evaporated to dryness using a vacuum rotary evaporator and re-dissolved with 3 mL of hexane. A spectrophotometer (UV-1650 PC Spectrophotometer, Shimadzu, Japan) at a wavelength of 450 nm was used to read the absorbance of extract. For the quantification of total carotenoid content, β-Carotene was used as a standard where it was diluted with hexane according to the concentration required. Total carotenoid content was calculated from the calibration curve and results were expressed as mg β-Carotene equivalent per 100 g fresh weight (FW) basis.

#### 3.4.2. Determination of Vitamin A

The determination of Vitamin A (beta-carotene) content was conducted using reverse-phase HPLC method [84]. The HPLC system was equipped with a Column C18- Reverse phase. The HPLC system was composed of acetonitrile: Methanol: Ethyl acetate (88:10:2) as a mobile phase at a flow rate 1.3 mL/min and the injection volume at 20µL. 10–20 g of sample was mixed with 95% ethanol (4 times volume of the weighted sample) and 20% potassium hydroxide (KOH) (volume equal to the weight of sample). Then, the refluxing process was carried out for 30 min after adding the boiling chips. The hydrolysate was extracted three times with 50 mL hexane. Then, the extract was washed with water and the washing step was repeated with anhydrous sodium sulfate. The hexane extract was then evaporated to dryness and re-suspended in the mobile phase. The extract was filtered using 0.45 nylon membrane filter before injected into the system. β-Carotene standard curve was used for quantification of vitamin A and the results were expressed as mg per 100 g dry sample.

#### 3.4.3. Determination of Vitamin C

AOAC’s official titrimetric method (method 967.21) was used for determination of vitamin C content in the sample [85,86]. The sample (10 g) was extracted using metaphosphoric acid (HPO_3_)-acetic acid (HOAc) solution (15 g of HPO_3_ and 40 mL of HOAc in 500 mL of deionized H_2_O). The extracts were centrifuged (3000 rpm for 15 min) and the supernatant was collected. l-ascorbic acid was used as a reference standard. Then, the samples and blank were titrated with indophenol reagent (dissolved 50 mg of 2,6-dichloroindophenol sodium salt and 42 mg of NaHCO_3_ into 200 mL with deionized H_2_O) to a light but distinctive rose pink (endpoint lasting ≥ 5 s). The ascorbic acid equivalent of samples was calculated using the formula below:

mg ascorbic acid per 100 g sample = X × A × V/Y × 100/W(1)

where, X: mL of indophenol used to titrate the sample, A: mL of indophenol used to titrate the ascorbic acid standard (equivalent to mg of ascorbic acid contained), V: total volume (mL) of sample used, Y: total volume (mL) of sample used in titration to pink color (10 mL), and W: weight of sample (g).

#### 3.4.4. Determination of Vitamin E

The determination of vitamin E was conducted based on AOAC method 971.30 [26]. Mixtures of two to 10 g of sample, 50 mL of 95% ethanol, 50% potassium hydroxide, and 0.25 g of ascorbic acid undergone refluxing process for 30 min at 40 °C. The solution was cooled and transferred into separating funnel by rinsing with 50 mL distilled water. After that, 25 mL of petroleum ether was added and shaken vigorously. Two-layers appeared and the upper layer (petroleum ether) was collected. The extraction step was replicated for two times. The ether extracts were pooled and washed with water until the solution became neutral. Anhydrous sodium sulfate was used to filtrate the washed ether extract. The ether extracts were then evaporated to dryness under N_2,_ diluted with methanol, and filtered again using 0.45 μm membrane filter. Then the sample was ready to be injected (10 μL) to reverse-phase HPLC-FLD system equipped with C18; 5 μm, reversed-phase column; 4.6 × 150 mm ID and a fluorescence detector at wavelength set 296 nm for excitation and 330 nm for emission. The mobile phase used was Methanol (A): Deionized water (B) (95:5) with flow rate 1.0 mL/min. The gradient column pump mode was set at 1–5 min 95% A, 5.5–6 min 97% A, 6–25 min 95% A. Peak were identified and quantified by developing standard curve with α-Tocopherol. The calculation of Vitamin E is based on the following formula:

E mg/kg = C × 10/Ws(2)

where, C: Concentration from the standard curve (ppm), Ws: Sample weight (g).

### 3.5. In Vitro Anti-Diabetic Assay

#### 3.5.1. Extraction

The sample was extracted according to method by Addai et al. [87]. A mixture of 1 g of sample with 10.0 mL of the solvent was stirred under magnetic stirring for two hours in the dark and at room temperature (27 °C). Then, the solutions were centrifuged for 15 min at 6000 rpm and the supernatant was collected. Then, the residue was washed using the 5.0 mL of the same solvent. The extract was shaken for 15 min and centrifuged again for 15 min at 6000 rpm. Then, the supernatant was pooled and dried using the rotary evaporator at 40 °C and further dried using the freeze dryer. Then the dried sample was diluted using the same solvent extraction at desired concentration for further analysis.

Three different solvent-water extraction systems were used (methanol, ethanol, and acetone) at 60 % *v*/*v* concentrations in distilled water and 100% distilled water. All analyses were performed in triplicate.

#### 3.5.2. *α*-Amylase Inhibition Assay

The antidiabetic activity of sample using *α*-amylase inhibition assay was performed following a starch-iodine test described as Kusano et al. [88] and Chakrabarti et al. [89] with slight modification whereby pre-incubation time increased, changed of concentration of enzyme and volume of iodine reagent used. Briefly, 20 µL of acarbose or plant extract at varying concentrations and 20 µL of 0.6 U/mL *α*-amylase (20 mM phosphate buffer saline, pH 6.9) was pre-incubated at 37 °C for 10 min. After pre-incubation, 40 µL of substrate solution was added to the reaction mixture and then further incubation at 37 °C for 15 min. The reaction was stopped by adding 80 µL of HCI (0.1 M). Then, 80 µL of iodine reagent (2.5 mM) was added and absorbance was measured at 630 nm. The positive control used were acarbose. The assay was carried out in triplicate in 96 well microplate.

The percentage inhibition was calculated using the formula:

% inhibition = {(A sample − A2)/(Ablank − Acontrol)} × 100(3)

where, A sample is the absorbance of the incubated mixture containing plant extract, starch, and amylase, A2 is the absorbance of incubating mixture of sample and starch, A blank is the absorbance of incubated solution containing starch, A control is the absorbance of the incubated mixture of starch and amylase. The IC_50_ value represents the concentration of inhibitor required to achieve 50% enzyme inhibition.

#### 3.5.3. *α*-Glucosidase Inhibition Assay

The enzyme inhibition activity against the alpha glucosidase was performed according to the modified method by Al-Zuaidy et al. [90]. Total volume of 100 µL reaction mixture contained 10 uL of samples at different concentrations, followed by the addition of 10 uL of 1U/mL alpha glucosidase enzyme and 70 uL 0.1 M phosphate buffer (pH 6.9). The contents were mixed and preincubated at 4 °C for 30 min. The reaction was initiated by the addition of 10 uL of 10 mM PNPG (*p*-nitrophenyl glucopyranoside). After incubation for 5 min at 37 °C, the absorbance of the yellow color produced was read at 405 nm using Elisa microplate reader (Biotek, EL800, Winooski, Vermont, USA). Acarbose and quercetin were used as positive control. All the enzyme, inhibitor and substrate solutions were made using the same buffer. The percentage of enzyme inhibition was calculated using the following formula;

% = [(absorbance control − absorbance sample)/absorbance control] × 100(4)

### 3.6. Screening and Identification of Bioactive Compounds Using LC-MS in the Ethanol Extract of Peel and Seed Kernel from M. odorata

#### 3.6.1. Sample and Standard Preparation

The sample preparation was carried out similar as extraction for anti-diabetic analysis. The sample concentration was prepared at 1 mg/mL. All the standards were mixed prepared with methanol (LC grade) at concentration of 10 ppm. Before injected into the system, the samples or standards were filtered using 0.2 µm nylon syringe filter. 

#### 3.6.2. Screening Untargeted Polyphenols that are Responsible as Anti-Diabetic Agents in the Peel and Seed Kernel of *M. odorata* Fruit

The LC-MS system consisted of an Agilent 1290 Infinity LC system coupled to Agilent 6520 Accurate-Mass Q-TOF mass spectrometer with dual ESI source (Agilent Technologies, Santa Clara, California, USA). The electrospray (ES) interface was used with the instrument operating in the positive and negative ion mode. Liquid chromatography was performed by injecting 1.0 µL of the sample (1 mg/mL) onto an Agilent Zorbax Eclipse XDB-C18, Narrow-Bore 2.1 × 150mm, 3.5 microns (P/N: 930990-902) (Thermo Fisher Scientific, Waltham, Massachusetts, USA). The column temperature was 25 °C. The chromatography system was operated in a gradient mode with a flow rate of 0.5 mL/min. The mobile phase consisted of 0.1% formic acid in water (Solvent A) and 0.1 % formic acid in acetonitrile (Solvent B) The gradient was increased linearly from 5% B over 0–5 min, then from 5% to 100% over 20–25 min. The total run time of the method was 30 min. 

Mass spectra were recorded in the negative and positive ionization mode over a mass range from *m*/*z* 100 to 3200.The MS scan source parameters were capillary voltage, 4000 V (positive ion) and 3500 V (negative ion), fragmentor voltage, 125 V and skimmer voltage, 65 V. The MS scan parameters were: nebulizer pressure and flow-rate on the nebulizer at 45 psi and 10 L/min, respectively, with a drying gas temperature of 300 °C. 

The MS data was processed through Masshunter Qualitative Analysis software (version B.05.00, Agilent Technologies, Waldbronn, Germany) which provides a list of possible molecules by using the Molecular Feature Extraction (MFE) according to the small molecule extraction algorithm with more than 100 counts of peak only. The inclusion of the compounds are the compound that have absolute height more than 5000 counts and relative height more than 2.5%. While, the ions species were allowed +H (hydrogen), +Na (sodium), +K (potassium), +NH4 (ammonium) for positive ion and H (hydrogen), −Cl (chloride) for negative ions. The closeness between molecular formula generated by the software and the real molecular formula of the compound are shown in the score (%). The highest the score the closer the similarity of molecular formula.

#### 3.6.3. Identification of Targeted Polyphenols in the Peel and Seed Kernel of *M. odorata* Fruit by Using UHPLC-ESI-Ortbitrap-MS/MS

For identification and confirmation of targeted compounds, 5 uL of sample and standard was injected to UHPLC-Ultimate 3000 system (Dionex, Sunnyvale, California, USA) was equipped with an autosampler and PDA detector. The separation was carried out on a column U-HPLC column (100 mm × 2.1 mm, 1.9 um, Hypersil Gold) (Thermo-Scientific, Waltham, Massachusetts, USA). The mobile phases were water 0.1% formic acid (A) in water and 0.1% formic acid in acetonitrile (B) at a flow rate of 0.4 mL min^−1^. The LC conditions were 5% B during 0–5 min, a linear increase from 5 to 55% B during 5–15 min, and finally from 55 to 5% B during 15–20 min. The PDA recorded spectra from 190 to 600 nm.

A Thermo Electron Q Exactive Focus-Orbitrap mass spectrometer equipped with a heated electrospray ion source (ThermoFisher Scientific, Waltham, Massachusetts, USA) and operated under Xcalibur 4.0 version software (Thermo Fisher Scientific, Waltham, Massachusetts, USA) that was used in negative ionization mode with the following conditions: spray voltage at 3200 V, capillary temperature and voltage at 320 °C, and tube lens voltage at 55 V. The sheath gas and auxiliary gas with a flow rate of 40 and 50 arbitrary units, respectively. Spectra were recorded in the range of *m*/*z* 50–7590 with a resolution of 70,000.

For identification purposes, two scan events were applied for the MS experiments. The first scan was in full scan MS mode and the second was data dependent scan that select the most intense ion or specified ions (accurate mass of authentic standard) in another setting from the first scan event for the acquisition of MS/MS spectra. The data dependent scan for MS/MS was confirmed with resolution at 17,500 and isolation window 2.0 amu. The collision-induced dissociation activation was set between 15, 30, and 50 V of collision energy. The instrument was calibrated using the manufacturer’s calibration standards every 7 days. The retention times, PDA spectra MS and MS/MS (fragment ions) analysis of the reference compounds and literature data, were used to identify the compound. The literature and several online databases (ChemSpider, Raleigh, North Carolina, USA) were also used for comparison of MS/MS spectra.

### 3.7. Statistical Analysis

Nutritional and anti-diabetic properties were carried out in duplicates or triplicates and data were reported as mean ± standard deviation. The differences of mean values among solvent systems and part of the fruits were determined using analysis of variance (ANOVA) followed by Tukey’s HSD tests at the significant level of *p* < 0.05. All statistical analyses were conducted using Minitab version 16.0 (Minitab Pty Ltd, Sydney, Australia).

## 4. Conclusions

The present work investigated the nutritional composition, anti-diabetic properties of different solvent extractions of peel and seed kernel from *M. odorata* fruit and the identification of their bioactive compounds. The seed kernel is a good source of CHO, protein, fat, ash, magnesium, phosphorus, and zinc. Meanwhile, peel was significantly high in potassium, sulphur, aluminum, calcium, manganese, iron, and boron. The peel also contains high amount of carotenoid, ascorbic acid, and α-Tocopherol. In this study, we provide evidences that the seed kernel possesses excellent *α*-amylase and *α*-glucosidase inhibitory activity. LC-MS screening data showed the presence of several compounds such as mangiferin, naringenin and isovitexin in peel, while beta-glucogaliin, theogallin, and 2-hydroxy-3,4-dimethoxybenzoic acid were found in the seed kernel. The excellent sources of minerals, vitamins, and fibers along with the potent anti-diabetic properties of peel and seed kernel revealed the potential of these fruit wastes to be used as functional ingredient in the food industry. It is also important to emphasize the promising ability of seed kernel as low-cost, valuable nutraceutical ingredients for diabetes management. However, comprehensive studies are required to elucidate the mechanisms associated with anti-diabetic properties and to identify their specific food applications as well as sustainability of the final output. To the best of the authors’ knowledge, this is the first report to demonstrate the nutritional and anti-diabetic potential *M. odorata* wastes. Further study could also focus on the efficacy and safety of the fruit wastes in animal and human models. 

## Figures and Tables

**Table 1:** Proximate composition of *M. odorata* peel and seed kernel.

Composition	% (Dry Weight)	
	Peel	Seed Kernel
Moisture	75.89 ± 0.70 ^a^	50.03 ± 0.98 ^b^
Crude protein	1.04 ± 0.04 ^b^	2.62 ± 0.06 ^a^
Crude fat	0.14 ± 0.04 ^b^	2.76 ± 0.06 ^a^
Ash	0.69 ± 0.01 ^b^	1.29 ± 0.07 ^a^
Carbohydrate (by difference)	22.25 ± 0.70 ^b^	43.31 ± 0.92 ^a^
**Fibre**		
Total Dietary Fibre	50.94 ± 7.19 ^a^	24. 75 ± 1.30 ^b^
Soluble	34.78 ± 3.73 ^a^	nd
Insoluble	16.16 ± 3.46 ^b^	24.75 ± 1.30 ^a^
**Sugar**		
Fructose	2.25 ± 0.38 ^a^	0.98 ± 0.05 ^b^
Glucose	0.83 ± 0.10 ^b^	1.08 ± 0.27 ^a^
Sucrose	2.66 ± 0.36 ^a^	2.66 ± 0.06 ^a^
**Energy (kcal)**	94.50 ± 2.12 ^b^	208.50 ± 4.95 ^a^

Values with the different letters in the same row are significantly different (*p* < 0.05). Mean ± SD (*n* = 2) except sugar, (*n* = 3). nd = not detected.

**Table 2:** Mineral Elements and Antioxidant Vitamins of *M. odorata* peel and seed kernel.

Parameters	Value (mg/100 g Dry Weight)	
	Peel	Seed Kernel
**Major mineral**		
Potassium	1203.01 ± 12.79 ^a^	875.69 ± 23.12 ^b^
Phosphorus	75.83 ± 0.34 ^b^	165.50 ± 3.39 ^a^
Calcium	391.83 ± 1.68 ^a^	141.48 ± 2.32 ^b^
Magnesium	147.75 ± 1.27 ^b^	166.88 ±4.49 ^a^
Sulphur	69.29 ± 3.59 ^a^	43.03 ± 1.38 ^b^
Sodium	10.77 ± 0.25 ^a^	10.56 ± 1.68 ^a^
**Trace mineral**		
Aluminium	0.76 ± 0.03 ^a^	0.27 ± 0.06 ^b^
Manganese	2.25 ± 0.03 ^a^	1.09 ±0.03 ^b^
Iron	1.36 ± 0.04 ^a^	1.08 ± 0.03 ^b^
Copper	0.65 ± 0.08 ^a^	0.78 ± 0.01 ^a^
Zinc	1.12 ± 0.03 ^b^	1.55 ± 0.01 ^a^
Boron	1.69 ± 0.01 ^a^	0.87 ± 0.00 ^b^
**Antioxidant vitamins**		
Total carotene content (TCC)	670.00 ± 30.00 ^a^	60.00± 0.00 ^b^
β-Carotene	100.75 ± 0.21 ^a^	0.05 ± 0.01 ^b^
Ascorbic acid	5.21± 0.00 ^a^	2.62 ± 0.00 ^b^
α-Tocopherol	0.99 ± 0.00 ^a^	0.21 ± 0.00 ^b^

Values with the different letters in the same row are significantly different (*p* < 0.05). Mean ± SD (*n* = 2–3).

**Table 3:** In-vitro anti-diabetic activity of different solvent extracts of *M. odorata* peel and seed kernel.

Parameters	Values (mg/mL)	
	Peel	Seed Kernel
***α*-Amylase inhibition activity**		
60% Acetone	33.72 ± 1.15 ^Aa^	5.81 ± 0.05 ^Bb^
60% Ethanol	9.96 ± 0.61 ^Ad^	3.44 ± 0.17 ^Bc^
60% Methanol	17.78 ± 0.23 ^Ac^	9.83 ± 0.28 ^Ba^
Water	26.27 ±1.32 ^Ab^	2.67 ± 0.05 ^Bd^
Acarbose	0.94 ± 0.05 ^D^	
***α*-Glucosidase inhibition activity**		
60% Acetone	10.52 ± 0.37 ^Ac^	0.29 ± 0.03 ^Bc^
60% Ethanol	38.28 ± 1.44 ^Ab^	1.75 ± 0.10 ^Bab^
60% Methanol	39.33 ± 0.91 ^Ab^	1.55 ± 0.10 ^Bb^
Water	59.45 ± 3.79 ^Aa^	2.10 ± 0.43 ^Ba^
Positive control	Acarbose (3.53 ± 0.59 ^D^) Quercetin (0.40 ± 0.02 ^E^)	

Results are expressed as IC_50_ mg/mL dry sample (mean ± standard deviation of three replicates, *n* = 3). Each type of solvent at concentration 60% *v*/*v* except water. Means followed by different uppercase letters in the same row are significantly different by Fisher test at 5% probability; means followed by different lowercase letters in the same column are significantly different by Fisher test at 5% probability.

**Table 4:** Polyphenols identified in the peel and seed kernel extract of *M. odorata* by LC-ESI-Q-TOF-MS.

Peak	*t*_R_ (min)	[M − H]^−^ (*m*/*z*)	[M − H]^+^ (*m*/*z*)	MF	Compounds	Part of the Fruits	Score
1	0.64	-	290.08	578.14	Apigenin 7-(2′′-*E*-*p*-coumaroylglucoside)	PL	75.62
2	0.65	-	381.08	358.09	Dihydrocaffeic acid-3-*O*-glucuronide	PL	89.51
3	0.69	-	193.07	192.06	Quinic acid	SK, PL	86.43
4	0.75	-	139.04	138.03	*p*-Salicyclic acid	PL	82.78
5	0.9	-	175.02	174.02	Dehydroascorbic acid	PL	96.55
6	1.4	331.07	350.11	332.07	Beta-Glucogallin	SK	95.99
7	2.2	343.07	345.08	344.07	Theogallin	SK	98.94
8	8.1	421.08	423.09	422.09	Mangiferin	PL	94.29
9	8.8	197.05	199.06	198.05	2-Hydroxy-3,4-dimethoxybenzoic acid	SK	97.95
10	8.86	-	273.08	272.07	(±)-Naringenin	PL	98.94
11	8.9	787.10	-	788.11	1,2,3,4-Tetragalloyl-alpha-d-glucose	SK	98.47
12	9.0	-	273.08	272.07	7,8,4′-trihydroxyflavanone	PL, SK	96.47
13	9.1	-	433.11	432.11	Isovitexin	PL	97.21
14	15.0	277.18	-	278.18	6-Paradol	SK	99.04
15	16.0	-	235.17	234.16	Curcumenol	PL, SK	94.74
16	23.86	401.31	-	402.31	1a,25-dihydroxy-24-norvitamin D3	SK	91.48

Peak, compound number; Rt, retention time (min); MF, molecular formula; score, percentage of the molecular formula generated by Masshunter software with the exact mass and the isotopic distribution. Part of the fruits, extracts where the compound has been identified; SK (seed kernel); PL (peel).

**Table 5:** Chromatogram and spectral properties of compounds detected in ethanol extracts of peel and seed kernel extract from *M. odorata* fruit by by LC-ESI-Orbitrap-MS/MS.

Peak	*t*_R_ (min)	[M − H]^−^ (*m*/*z*)	MF	Fragment Ions MS^2^ (*m*/*z*)	Compounds
1	1.44	169.01	170.12	67.0 (28), 124.0 (24), 125.0 (100)	Gallic acid
2	7.23	289.07	290.20	57.0 (30), 83.0 (20) 93.0 (20), 97.0 (24), 109.0 (100), 123.0 (70), 137.0 (28), 151.0 (14), 203.0 (14), 245.0 (18), 289.1 (30)	Catechin
3	8.26	197.04	198.17	125.0 (100), 140.0 (24), 169.0 (74), 197.0 (60)	Ethyl gallate
4	8.28	289.07	290.20	57.0 (36), 69.0 (24), 81.0 (24), 97.0 (30), 109.0 (100), 123.0 (74), 137.0 (30), 151.0 (30), 203.0 (30), 245.0 (34), 289.0 (30)	Epicatechin
5	8.34	421.07	422.33	258.0 (58), 259.0 (50), 271.0 (66), 272.0 (60), 301.0 (100), 331.0 (70), 421.7 (38)	Mangiferin
6	8.66	162.03	163.03	65 (16), 93 (50), 119 (100)	*p*-Coumaric acid
7	9.60	300.99	302.01	117.0 (22), 145.0 (28), 173.0 (12), 200.0 (10), 229.0 (10), 283.9 (10), 300.9 (100)	Ellagic acid
8	10.49	317.03	318.20	63.0 (48), 65.0 (66), 83.0 (30), 107.0 (44), 109.0 (100), 137.0 (76), 151.0 (76), 317.0 (24)	Myricetin
9	12.56	269.54	270.20	65.0 (24), 117.0 (100), 151.0 (10), 269.0 (42)	Apigenin
10	12.60	285.04	286.20	65.0 (14), 93.0 (24), 117.0 (18), 143.0 (10), 161.0 (10), 187.0 (5), 211.0 (10), 239.0 (5), 285.04 (100)	Kaempferol

Peak, compound number; Rt, retention time (min); MF, molecular formula; fragment ions, the product ions produced from MS/MS full scan.

**Table 6:** Relative Abundance Values of TIC Peaks of Peel and Seed kernel from *M. odorata*.

Peak	*m*/*z*	Peel	Seed Kernel
1	169.01	5.84	100.00
2	289.07	100.00	90.21
3	197.05	18.63	100.00
4	289.07	100.00	nd
5	421.08	100.00	2.79
6	163.04	36.92	nd
7	300.99	5.38	3.70
8	317.03	nd	6.37
9	269.05	19.12	32.42
10	285.04	5.13	6.81

* nd = not detected.

## Supplementary Materials

The following are available online, Figure S1: Standard chromatogram of polyphenols by LC-MS/MS, Figure S2: Sample (Peel (A) and Seed kernel (B)) chromatogram of single polyphenol detected by LC-MS/MS., Figure S3: Molecular structure of compounds in the peel and seed kernel extract of *M. odorata* that were detected during screening using LC-MS (on the left), and identified using LC-ESI-Q-TOF-MS (on the right).
