# Supplementary material for: Nutritional Composition, Anti-Diabetic Properties and Identification of Active Compounds Using UHPLC-ESI-Orbitrap-MS/MS in Mangifera odorata L. Peel and Seed Kernel
Source: Molecules. 2019 Jan 16;24(2):320. doi: 10.3390/molecules24020320 (PMC6359265; doi:10.3390/molecules24020320)
Supplement: Supplementary file 1 [file molecules-24-00320-s001.pdf]

Supplementary Materials:

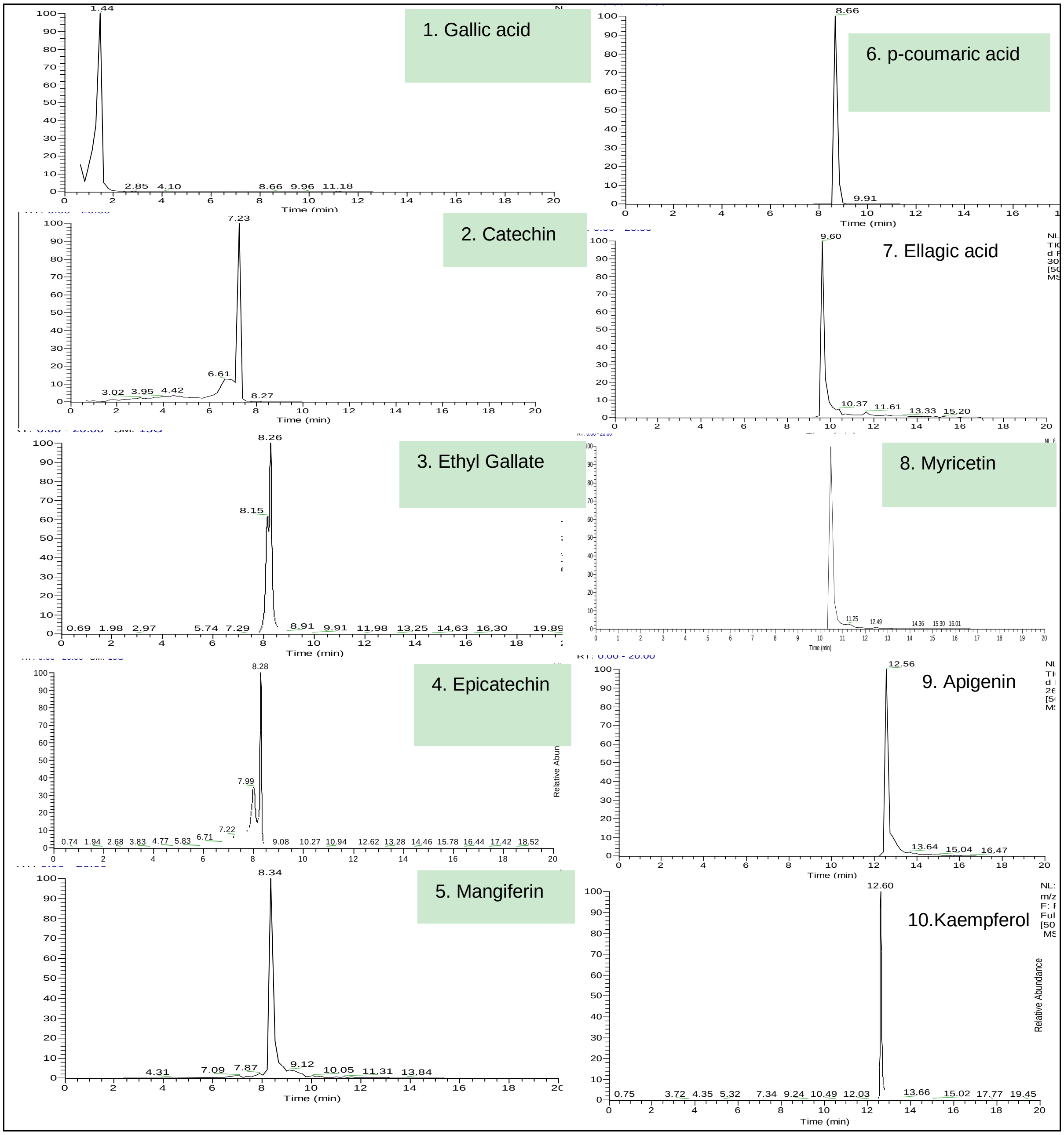

Figure S1. Standard chromatogram of polyphenols by LC-MS/MS

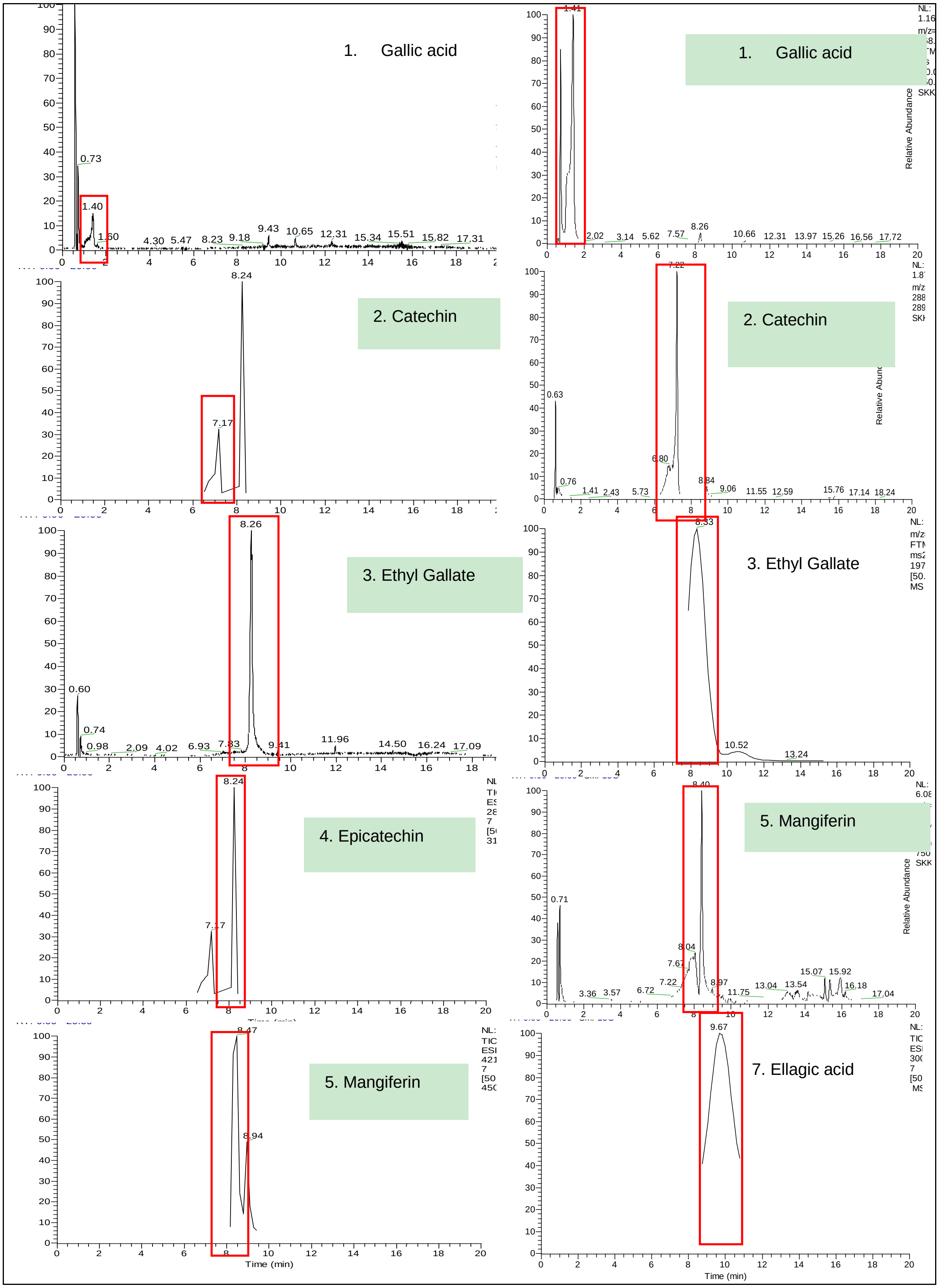

1. Gallic acid
1. Gallic acid
2. Catechin
2. Catechin
3. Ethyl Gallate
3. Ethyl Gallate
4. Epicatechin
5. Mangiferin
5. Mangiferin
7. Ellagic acid
Relative Abundance
Time (min)
Time (min)

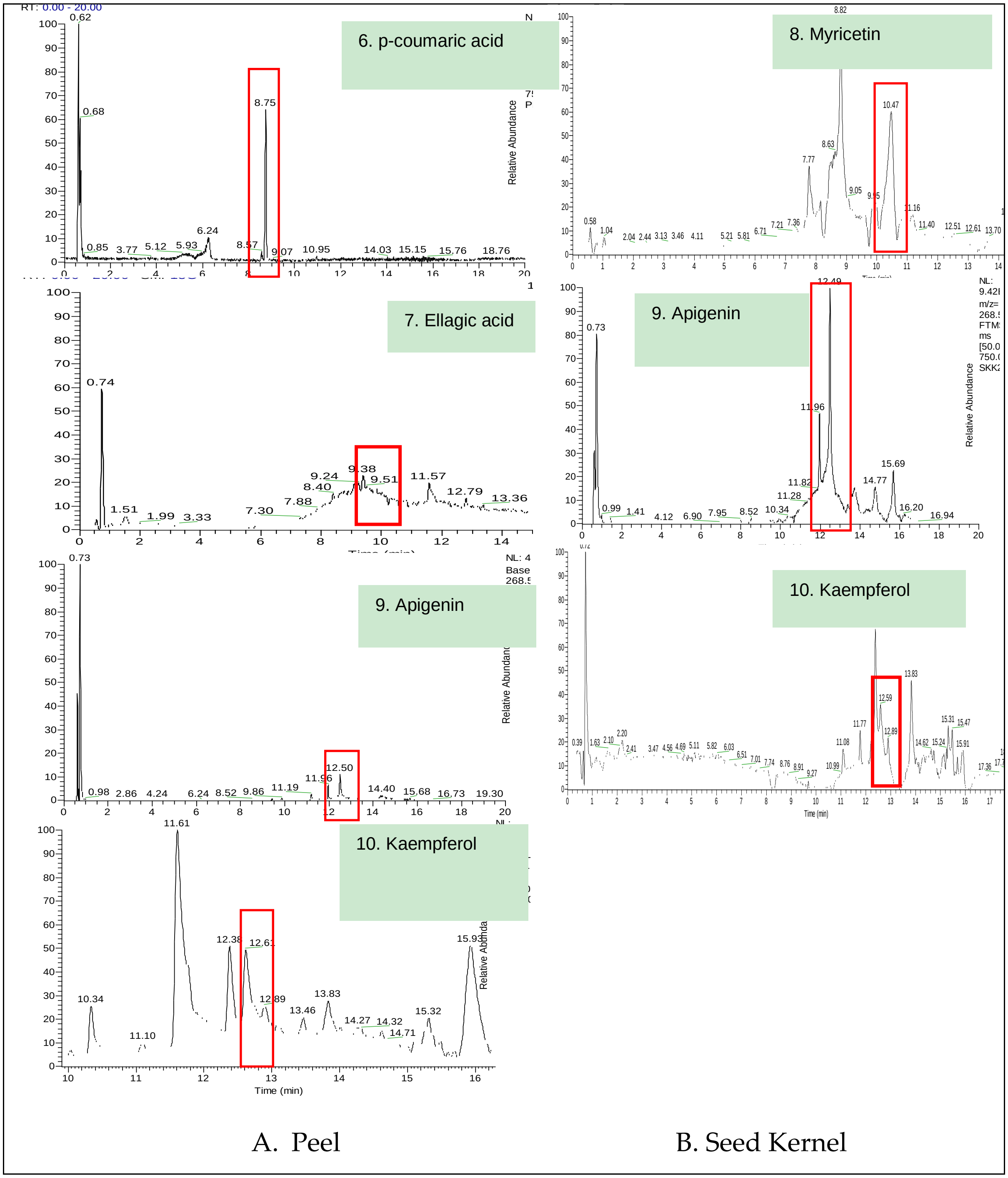

Figure S2. Sample (Peel (A) and Seed kernel (B)) chromatogram of single polyphenol detected by LC-MS/MS

Apigenin 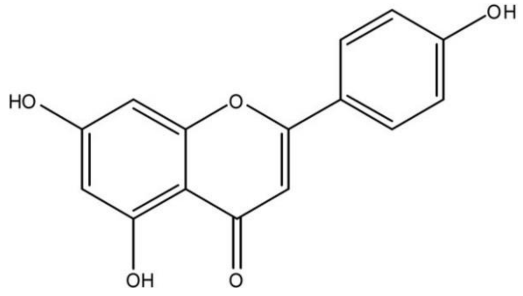	Gallic acid 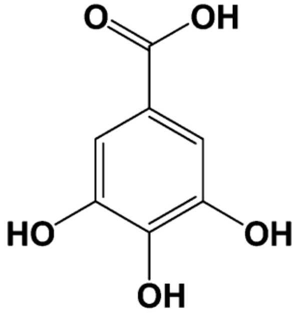
Dihydrocaffeic acid-3-O-glucuronide 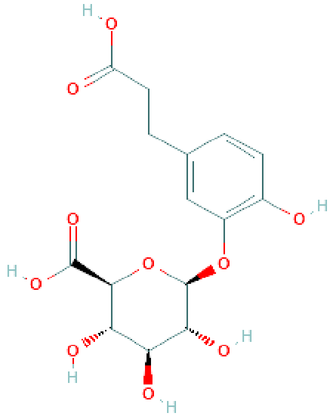	Catechin 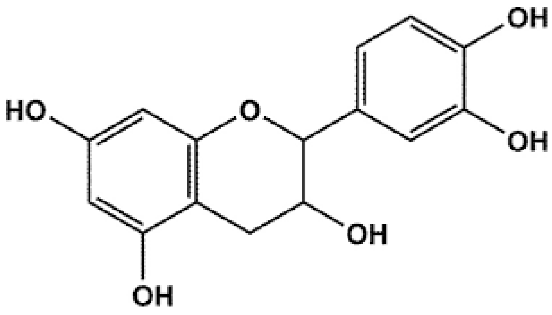
Quinic acid 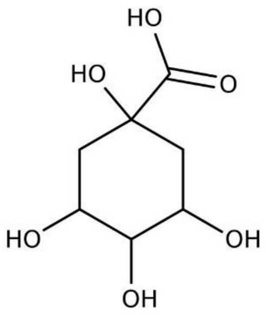	Ethyl gallate 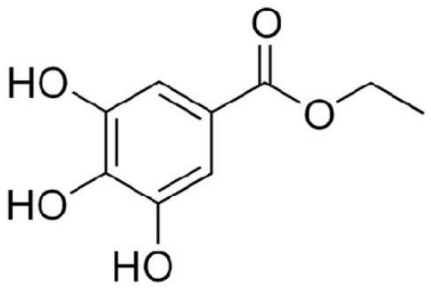
ρ-salicyclic acid 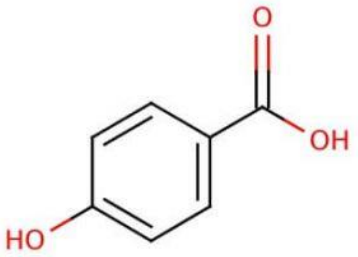	Epi-catechin 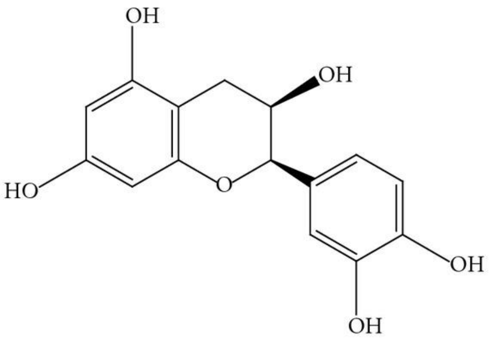

Dehydroascorbic acid 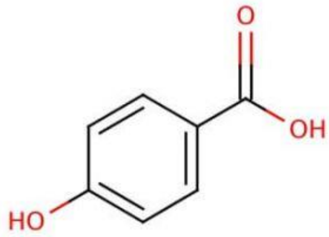	Mangiferin 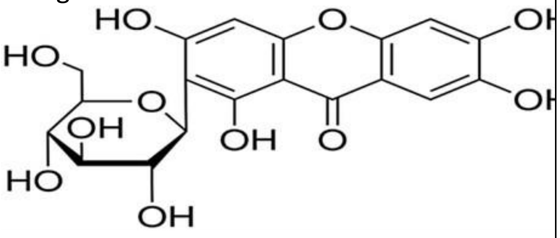
Beta- glucogallin 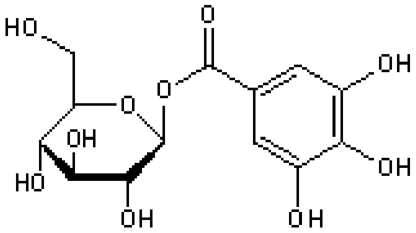	ρ-coumaric acid 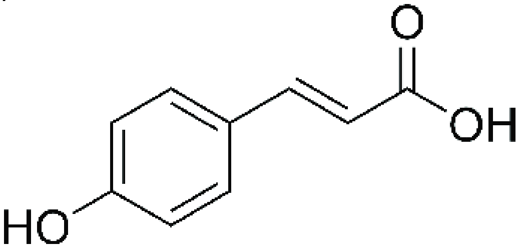
Theogallin 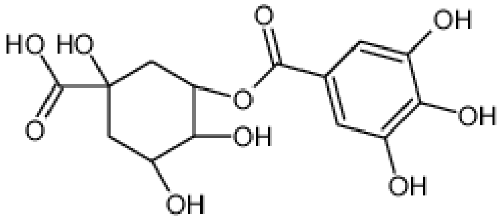	Ellagic acid 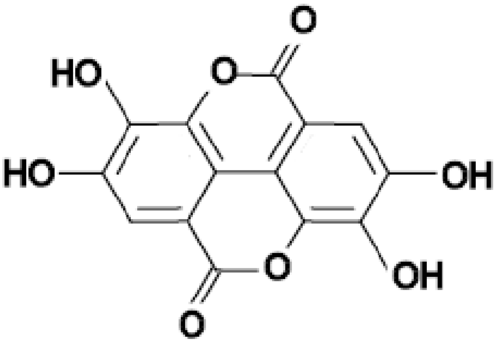
Mangiferin 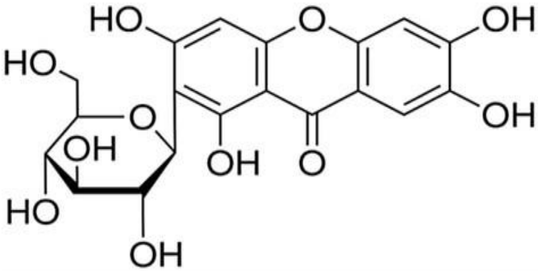	Myricetin 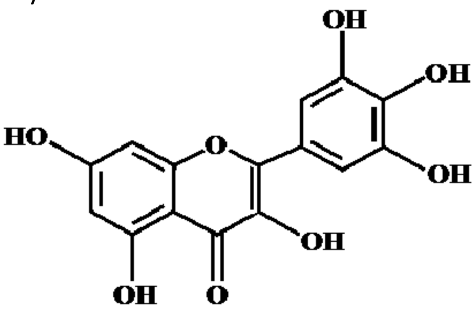

2-Hydroxy-3,4-dimethoxybenzoic acid<br>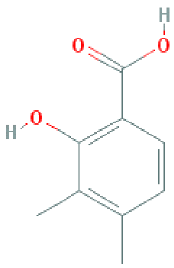	Apigenin<br>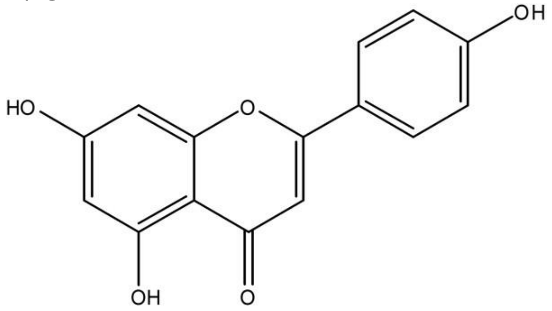
Naringenin<br>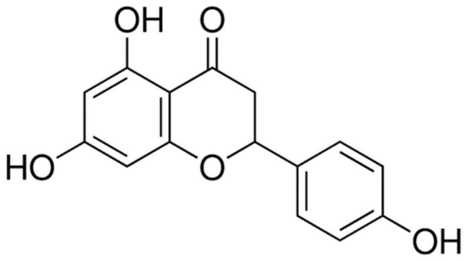	Kaempferol<br>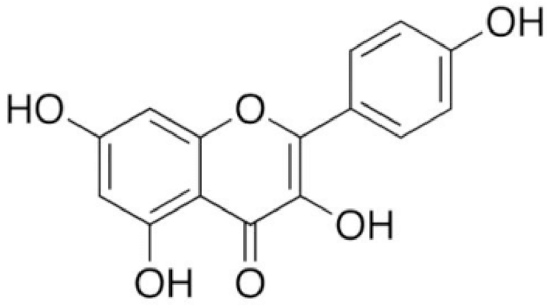
1,2,3,4-Tetragalloyl-alpha –D-glucose<br>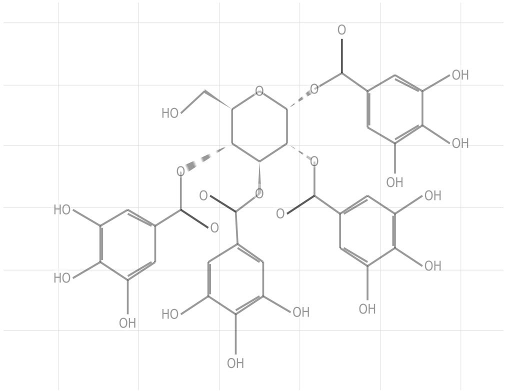	
7,8,4’-trihydroxyflavanone<br>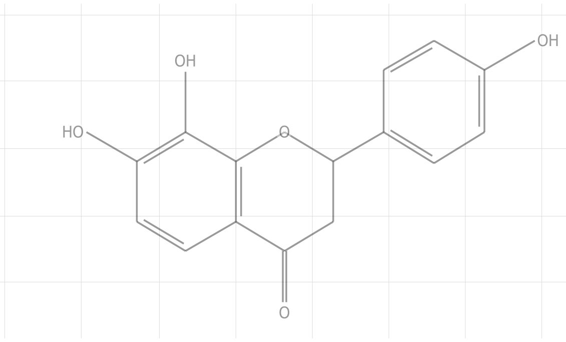	

Isovitexin<br>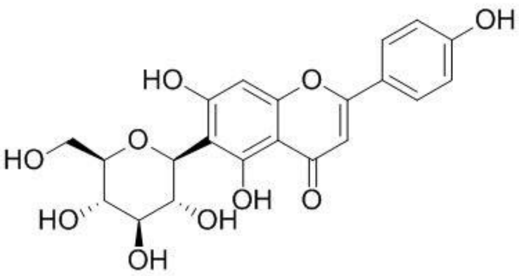<br>6-Paradol<br>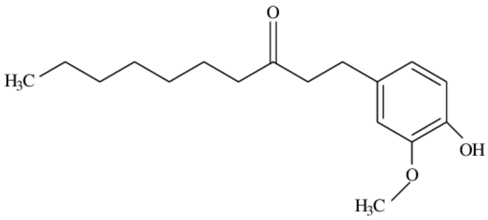<br>Curcumenol<br>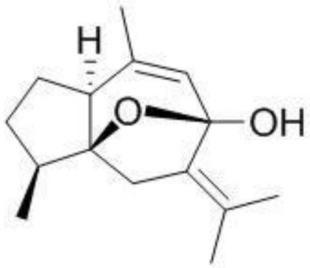<br>1a,25-dihydroxy-24-norvitamin D3<br>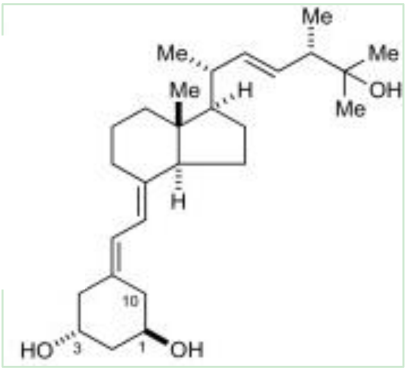	

Figure S3. Molecular structure of compounds in the peel and seed kernel extract of *M. odorata* that were detected during screening using LC-MS (on the left), and identified using LC-ESI-Q-TOF-MS (on the right).
